# Anemia in lupus nephritis; etiological profile

**DOI:** 10.12861/jrip.2013.32

**Published:** 2013-09-01

**Authors:** Mohammad-Reza Ardalan

**Affiliations:** ^1^Chronic Kidney Disease Research Center, Tabriz University of Medical Sciences, Tabriz, Iran

**Keywords:** Erythropoietin, Systemic lupus erythematous, Anemia

**Implication for health policy/practice/research/medical education:**

Erythropoietin treatment could augment the immune response and could be a tiger for systemic lupus erythematous flare-up and increasing the activity of renal and systemic lupus.

Anemia is a common clinical finding in end-stage renal failure (ESRD). Systemic lupus erythematous (SLE) is an important cause of chronic kidney disease and anemia in lupus nephritis has different and complex causes, including; anemia of chronic disease (ACD), autoimmune hemolytic anemia (AHA), iron deficiency anemia (IDA), pure red cell aplasia (PRCA), pernicious anemia (PA), myelofibrosis, hemophagocytic histiocytic syndrome, thrombotic microangiopathy and drug induced myelotoxicity (1). Combination of lupus nephritis and lupus-induced bone marrow fibrosis create a difficult situation to make a decision between saving the kidney and preventing future myelosuppression.

We recently admitted a 23 year-old-female with advance renal failure and oliguria. Physical examination on admission revealed a blood pressure of 130/90 mmHg and pulse rate of 105 minute/min. Cardiac examination showed a loud systolic murmur. Abdominal examination disclosed a palpable spleen. Hemodialysis was started with temporary jugular vein catheter. Laboratory examination revealed; hemoglobin: 4.1 g/dl, hematocrit: 17%, MCV: 78.6 fl, MCH: 24.4 pg/cell and MCHC: 31.1 g/dl. White blood cell count: 3.6×10^3^/µl (neutrophil: 55.9%, lymphocyte: 30.4%, monocyte: 10.5%, eosinophil: 3.7%, basophil: 0.5%) and reticulocyte was 1%. Also, platelets count; 156×10^3^/µl, serum creatinine: 7.63mg/dl, BUN: 84 mg/dl, uric acid: 7.5 mg/dl, phosphorous: 5.8 mg/dl, calcium: 8.4 mg/dl. Serum complements were as follow: C3: 68 mg/dl (90-180), C4: 10 mg/dl (10-40) and CH50: 76% (90-98). Further laboratory tests revealed; anti MPO–ANCA (IgG): 0.8 u/ml (<12; ELISA), Anti PR3-ANCA(IgG): 6.0 U/ml (<12; ELISA), Anti-dsDNA: 42 U/ml( <12; ELISA), LDH: 800 IU/ml (< 500 ), AST: 36 IU/ml, ALT: 38 IU/ml (<40), serum Na: 139 meq/l, serum K: 5, 0 meq/l, total bilirubin: 0.33 mg/dl (0.1-1.2), direct bilirubin: 0.09 mg/dl (0.1-0.4). Antiphospholipid antibody (IgG/IgM) was negative. Stool examination was negative for occult blood. Also upper gastrointestinal endoscopy had normal results. Peripheral blood examination revealed a large number of teardrop red blood cells. Bone marrow aspiration was unsuccessful (dry tap), bone marrow biopsy was compatible with myelofibrosis. Renal allograft biopsy revealed diffuse lupus nephritis (lupus nephritis class IV) with cellular crescents. This situation was difficult to make a decision to start an intensive immunosuppressive therapy in the presence of marrow failure. Despite receiving adequate dialysis and blood transfusion and adjusted immunosuppressive therapy her general condition worsened and died two months after the admission.

In SLE, there is an association between the level of inflammatory cytokines and intensity of anemia. Inflammatory cytokines inhibit erythropoietin production and erythroid progenitor cells proliferation. In severe cases, inflammatory cytokines lead to myelofibrosis (1,2). Renal parenchymal infiltration by inflammatory cells could directly inhibit renal erythropoietin production (1). Thrombotic thrombocytopenic purpura (TTP) or Coombs’ negative, microangiopathic hemolytic anemia occur in 2%–3% of SLE patients (1,3). Autoimmune hemolytic anemia occurs in 5–10% of patients. It is associated with low complement level, presence of anti-dsDNA antibodies and elevated IgG anti-cardiolipin antibody that act as an anti-red blood cell antibody (1,2). Positive coombs test without actual hemolysis could happen in up to 65% of SLE patients (1,2). Gastrointestinal blood loss because of long standing corticosteroid therapy is a very common cause of iron deficiency anemia in SLE patients (1). Hematopoietic progenitor cells are targeted by autoantibodies and create various clinical syndromes including: aplastic anemia, thrombocytopenia, pure red cells aplasia, bone marrow hypo-cellularity and fibrosis (1,4,5). Erythropoietin (EPO) also has its special challenge in lupus nephritis. Indeed, inadequate EPO production and EPO resistance are contributor of anemia in SLE (1). Anti-EPO antibodies are found in patients with active disease with low serum complement levels (1,6). These antibodies are similar to those that rarely developing after exogenous EPO therapy. Immunosuppressive treatment could improve the anemia by suppressing these autoantibodies. Anti-EPO antibodies not only block the biologic activity of EPO but also interfere with EPO measurement (1,6). There are some rations against widespread use of EPO in SLE patients. Potentially it could be associated with anti-EPO antibodies that could inhibit the action endogenous EPO (2,3,6). EPO treatment could augment the immune response and could be a tiger for SLE flare-up and increasing the activity of renal and systemic lupus (1,4-6).

## Author’s Contribution

MRA is the single author of this paper.

## Fund/Support

No financial support by any institution.

## Conflict of interests

None to declare.
